# The Influence of Obesity on Patient Reported Outcomes following Total Knee Replacement

**DOI:** 10.1155/2012/185208

**Published:** 2012-10-17

**Authors:** Vandana Ayyar, Richard Burnett, Fiona J. Coutts, Marietta L. van der Linden, Thomas H. Mercer

**Affiliations:** ^1^Rehabilitation Sciences, Queen Margaret University, Queen Margaret University Drive, Musselburgh, East Lothian, EH21 6UU, UK; ^2^Orthopaedic Department, Royal Infirmary of Edinburgh, 51, Little France Crescent, Old Dalkeith Road, Edinburgh, Midlothian, EH16 4SA, UK

## Abstract

This study retrospectively analysed the effects of obesity as described by Body Mass Index (BMI) on patient reported outcomes following total knee replacement. Participants (105 females and 66 males) who had undergone surgery under the care of a single surgeon were included in the review and were grouped according to their preoperative BMI into nonobese (BMI < 30 kg/m^2^), (*n* = 73) obese (BMI ≥ 30 kg/m^2^) (*n* = 98). Oxford Knee Score (OKS) and Short Form 12 scores (SF12) were taken preoperatively and 6 and 12 months after surgery to analyse differences between groups in the absolute scores as well as changes from before to after surgery. Preoperatively, the obese group had a significantly poorer OKS compared to non obese (44.7 versus 41.2, *P* = 0.003). There were no statistically significant group effects on follow-up or change scores of the OKS and SF12. Correlations coefficients between BMI and follow-up and change scores were low (*r* < 0.201). There were no significant differences in the number of complications and revisions (local wound infection, 6.7% non obese, 11% obese, postoperative systemic complication, 8% non obese, 12% obese, revision, 4% nonobese, 3% obese). In conclusion, our findings indicate similar degrees of benefits from the surgery irrespective of patient BMI.

## 1. Introduction

Total knee replacement (TKR) is a widely performed surgical operation and has shown to have a high success rate in improving the function and quality of life in patient with arthritis of the knee [1, 2]. The number of knee replacements performed in Scotland has been rising steadily since 1992. In 2009, 6884 primary knee replacements were performed in Scotland [3]. The high incidence of TKR is consistent with the increasing obesity in the population and the association of obesity with the onset and progression of knee arthritis [4].

Obesity has been defined according to the Body Mass Index (BMI), where a BMI above 30 kg/m^2^ is considered obese [5]. It has been seen that BMI increases with age reaching a peak incidence in the age group of 60–69 years coinciding with the average age range for primary joint replacement [4]. The global increase in the prevalence of obesity in the total knee arthroplasty population has lead to concerns regarding the outcomes of the surgery in obese patients. Obesity is considered as a risk factor for a number of surgical complications and there also remain concerns about the impact of the added stress on the underlying bone and implant material in obese patients thereby affecting the prosthetic longevity and functional gain [6–8]. The question whether obese total knee arthroplasty patients are predisposed to adverse outcomes has been researched previously with conflicting results. On defining obesity as BMI greater than 30 kg/m^2^, several studies reported no significant difference in the outcomes in obese and nonobese patients [9–14], while other studies show obese patients with inferior outcomes in terms of postoperative complications, function, and revision rates [15–18]. 

Most of the previous literature on obesity in TKR is centred on surgical aspects and surgeon/investigator measured outcomes. The Knee Society Score (KSS) has been most commonly used outcome measure in these studies [6, 9–13, 16–19]. A few studies assessing the impact of obesity on total knee replacement outcomes have focused on the use of patient administered outcomes. Patients' perception of their functional difficulties specific to their health problem and their perception of their general quality of life can provide a complete evaluation of their perceived benefits of the intervention. While some studies assessing outcomes in obese total knee arthroplasty patients have assessed various self-report outcomes measuring the quality of life [12, 20, 21], only a few have assessed self-report questionnaires specific to knee function such as the Western Ontario and McMaster Universities Arthritis Index (WOMAC) [14, 22, 23] and none have assessed self-reported knee function using the Oxford Knee Score (OKS).

Furthermore, most previous studies have assessed patients from the databases or registers of different surgeons, adding to the heterogeneity of the sample and thus making it more difficult to find an effect of obesity.

Therefore, the purpose of this retrospective epidemiological evaluation is to assess the influence of Body Mass Index (BMI) on the patient perceived outcomes of TKR in patients undergoing total knee replacement under the care of a single surgeon. The current study assesses patients' perception of their functional ability specific to their knee replacement, as measured by Oxford Knee Score [24] and also their quality of life as measured by Short Form 12 questionnaire (SF12) [25].

## 2. Material and Methods

### 2.1. Participants

Data of 171 patients who underwent primary total knee replacement (160 with osteoarthritis and 11 with rheumatoid arthritis) at a single hospital between January 2005 and December 2008 were available for review for this retrospective study. Of the 171 cases (73 nonobese and 98 obese) for which baseline and complications data were available, analyses of follow-up scores were done for 64 cases (25 nonobese and 39 obese) with complete follow-up data. All patients were operated using a similar surgical approach by a single surgeon with a medial parapatellar approach. Kinemax plus or Triathlon total knee replacements were used. Postoperative care and rehabilitation in the hospital are based on an integrated care pathway and were identical for all patients.

### 2.2. BMI Groups

Body Mass Index (BMI), routinely recorded for all patients at the time of their preoperative assessment, was extracted for the study from the preoperative case notes of the patients. BMI is defined as the body weight in kilograms divided by the square of the height in meters. Using the World Health Organization definition of obesity, patients were divided into BMI groups of nonobese (BMI < 30 kg/m²) and obese (BMI ≥ 30 kg/m²).

### 2.3. Outcome Measures

The hospital database records information on self-report questionnaires of patients who are admitted to the hospital for elective orthopaedic surgery. For total knee replacement surgery, the Oxford Knee Score and Short Form 12 are administered routinely to the patients approximately one week prior to surgery, six months after surgery, and one year after surgery. 

Oxford Knee Score [24] is an assessment tool specific to the total knee replacement. It has been tested by its authors for internal consistency, test retest reliability, its construct validity with Knee Society Score, SF36 and Health Assessment Questionnaire (HAQ), and sensitivity to change [24]. It consists of 12 equally weighted questions addressing patients' assessment of their knee function and its effects on their quality of life. Each question is scored from 1 to 5 with a minimum total score of 12 indicating the least difficulty and a maximum score of 60 indicating most functional difficulties. 

The SF12 questionnaire [25] is an instrument used to measure overall physical and mental health; it also consists of 12 questions. The questionnaire is an adaptation of the Short Form 36 (SF 36), showing close and linear relation with the SF-36 [25]. The total scores are shown as two meta scores, a physical component summary (PCS), and a mental component summary (MCS). The lowest score is 0, indicating the worst possible health and the highest score is 100, indicating the best possible health.

### 2.4. Ethics

Ethical approval for the study was granted by the local hospital trust research ethics and university ethics committees.

### 2.5. Primary Data Analysis

Data was screened for normality using the Kolmogorov Smirnov test. Differences between the groups in patient demographics and preoperative outcome measures were analysed using one-way ANOVA and Fisher Exact tests. Both postoperative scores and the differences between preoperative and follow-up scores were analysed for any effects of BMI classification. Differences between preoperative and follow-up scores (change scores) were calculated from absolute scores by subtracting the baseline scores from the scores at the two follow-up assessments from baseline. This means that for OKS, a negative change score describes an improvement, while, for SF12 components, a positive change score indicates an improved quality of life. Between group comparisons of the pre- and postoperative scores, which were not normally distributed, were carried out using Kruskal-Wallis test (BMI group effect) and Friedman's ANOVA (time effect). Change scores which were normally distributed were analysed using one-way ANOVA to compare the improvement in outcome scores at 6 months and one year between the two BMI groups. A Bonferroni correction was applied for multiple comparisons.

Spearman correlation coefficients between BMI and absolute and change scores were also calculated.

The level of significance was set at *P* < 0.05 for all statistical tests. All statistical analyses were carried out using SPSS version 19.0.

## 3. Results

### 3.1. Baseline between Group Comparisons

The preoperative patient characteristics of the two BMI groups were as shown in Table 1. The obese group consisted of more females (48.0% versus 69.7%, *P* = 0.002). Further, more patients in the obese group suffered from hypertension (72.2 versus 45.3%, *P* < 0.001) and Diabetes Mellitus (18.3 versus 6.6%, *P* = 0.015). Age was lower in the obese group but this did not reach statistical significance (65.0 versus 65.7 years old, *P* = 0.089). The Oxford Score at the preoperative assessment was significantly higher, which means higher functional difficulties in the obese group compared to the nonobese group (44.7 versus 41.2, *P* = 0.003). Preoperative SF12 components were not significantly different between the two groups.

Average duration of hospital stay was similar for both groups; 6.5 days versus 6.7 days for the nonobese and obese group, respectively.

### 3.2. Within Group Comparison of Absolute Scores, Time Effect

Absolute scores for the three BMI groups are given in Table 2. Physical function, as measured by OKS and the physical component of the SF12 showed significant improvement from preoperative to both follow-up assessments (*P* < 0.001). However, the mental component of the SF12 did not show any significant difference between the three assessment points (*P* = 0.436). For the OKS and the SF12 physical component the biggest improvement was seen from pre-surgery to 6 months with little or no change from 6 months to one year. This was the case for both BMI groups.

### 3.3. Between Group Comparison

Postoperative scores are given in Table 2. There were no statistically significant differences between the two groups in any of the postoperative outcome measures. This was also the case when the analyses were repeated with the preoperative value as a covariate.

### 3.4. Between Group Comparison of Change Scores

The mean and standard deviations for change scores for the two BMI groups are given in Table 3. On comparison between groups using change in OKS scores, no significant difference was seen between the BMI groups for change in OKS from preoperative to 6 months followup or from preoperative to one year followup. Similarly no significant difference was observed between groups for change scores (preoperative to 6 months followup, or preoperative to one year follow up) for both physical and mental component of SF12.

### 3.5. Correlations between Outcomes and BMI

Table 4 shows the strength of the relationship between the outcome measures and BMI value. Only the relationship between preoperative OKS and BMI showed a weak but statistically significant correlation (*r* = 0.271, *P* < 0.001) indicating a higher score, hence more functional difficulty with increasing BMI value. However, the change scores at 1 year show a negative correlation with BMI (*r* = −0.194, *P* = 0.086) indicating that those with higher BMI values were improving slightly more.

**Figure 1:**
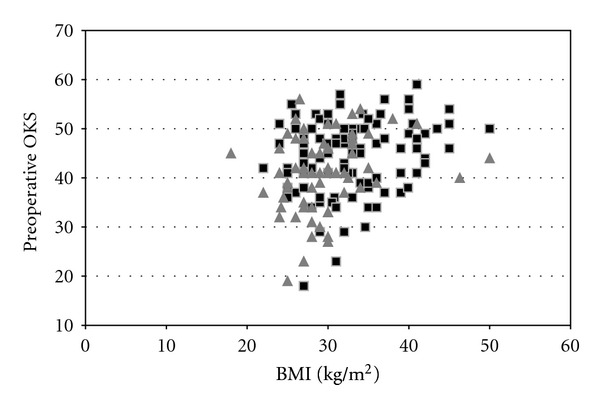
Scatter plot showing a relationship between BMI and preoperative OKS. Black squares indicate female patients and the grey triangles indicate male patients.

**Figure 2:**
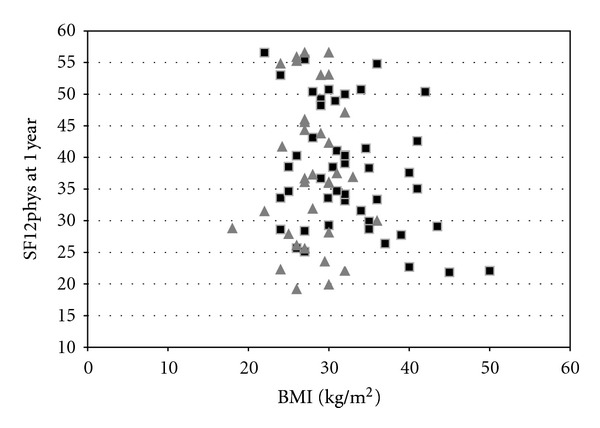
Scatter plot showing a relationship between BMI and SF12 (PCS) at one year after surgery. Black squares indicate female patients and the grey triangles indicate male patients.

Figures 1 and 2 show scatter diagrams of the outcomes which showed the strongest relationship with BMI; preoperative OKS (*r* = 0.271); the physical component of the SF12 at one year (*r* = −0.206, *P* = 0.061), the latter possibly indicating a trend toward a worse physical quality of life with increasing BMI. Data of female and male participants have been displayed separately.

### 3.6. Complications and Revisions

A total of 16 patients had a local complication (wound infection or wound leak) after surgery, of which, 5 (6.7%) were from the nonobese and 11 (11%) were from the obese group (Table 5). A total of 18 patients had a postoperative systemic complication of which 6 (8%) were from nonobese and 12 (12%) from the obese group. A total of 6 (3.5%) patients underwent a revision surgery after the primary total knee replacement of which 2 (3%) were in the nonobese and 3 (3%) in the obese group. The rate of the above complications and revision was not statistically significantly different between the two groups.

## 4. Discussion

The role of BMI on TKR outcomes has been a topic of much debate. Concerns over the adverse effects of higher BMI on the results of the surgery have led to some primary health care trusts using BMI > 30 as a cut-off point for screening patients for total knee replacements. The current study aimed to assess the influence of obesity as categorized by BMI on the patient reported outcomes after total knee replacement. We found that at short term (up to one year after surgery), the patient perceived benefits of total knee replacement were not different among two groups with different BMI. 

It has been reported that most improvement in function after total knee replacement occurs up to 26 weeks after which little improvement is gained [26]. The same was seen for our sample, with improvement in knee related function from the preoperative state to postoperative state (6 months and one year) significant in all BMI groups of the study, while, there was little change in knee function from 6 months postoperatively to one year postoperatively. This again, was true for both BMI groups.

The analysis of the self-report measures was carried out with both the absolute scores and the change scores. While the preoperative absolute score for knee function was poorer for the obese compared to the other groups, we found no significant difference in the 6 month and one year postoperative scores between any of the groups. The change in knee function and quality of life from before to 6 month and one year after surgery which was analyzed using change scores was also not significantly different between the groups.

Correlation analysis both numerically and as shown graphically in the scatter diagram indicated no or weak relationships between BMI values and both postoperative scores and change scores.

Finally, the rates of complications and revisions were not statistically significant between the two BMI groups. Therefore, our findings indicate similar degrees of benefits as quantified by the OKS and SF12 and complications and revision rates from the surgery irrespective of patient BMI.

The findings of our study are consistent with that of the majority of other studies investigating the effect of BMI on another patient reported outcome, the Western Ontario McMaster University Osteoarthritis Index (WOMAC) [14, 22, 23]. Nunez et al. [23], defining their BMI categories as those with a BMI > 35 (*n* = 60) for severely obese and those with a BMI < 35 (*n* = 60) as the control group, observed similar significant improvement in both groups for the total WOMAC at 12 months after operation. A larger study by Stickles et al. [14] saw no significant difference in change scores for WOMAC between their five BMI groups (BMI < 25, *n* = 146, BMI = 25–30, *n* = 304, BMI = 30–35, *n* = 271, BMI = 35–40, *n* = 149, and BMI > 40, *n* = 92) at a one year followup. In a separate rating of stair ascending and descending difficulty and satisfaction with surgery, Stickles et al. [14] concluded that despite finding greater difficulty with stairs, obese patients were as satisfied with the results of the surgery as other patients.

Even at longer followup of 5–11 years, no difference in the improvement in WOMAC scores between BMI groups (BMI < 25, BMI = 25–30, BMI = 30–35, BMI = 35–40, and BMI > 40) [22]. Contrary to these findings, Hawker et al. [27] in a community based study found that though BMI was not a significant predictor of pain, higher BMI was associated with worse physical function on the WOMAC at 2–7 years after surgery in 2 of their 3 stratified samples (*P* = 0.02 and *P* = 0.01).

The conflicting results in studies with midterm to longer term followup are further seen in studies assessing investigator measured outcomes such as the Knee Society Score (KSS). KSS and radiographic outcomes were not found to be statistically different between obese (BMI > 30) and nonobese (BMI < 30) at a followup ranging from 5 to 6 years [9, 13]. In contrast, other midterm to long term studies have observed a poorer outcome in obese (BMI > 30) [16–18]. Foran et al. [16, 17] in two case control studies with a followup of approximately 7 years and 15 years saw lower postoperative KSS scores and less improvement of scores from pre- to postoperative in the obese compared to the obese. Griffin et al. [18] also saw poor KSS (function component of KSS) in the obese at a mean follow-up period of 10.6 years.

Change in quality of life as assessed by the physical and mental component score of SF12 in this study was also similar in all BMI groups in our study. Other studies using SF 36 [14, 21] or SF12 [22] also saw no effect of BMI on this measure.

This study had several limitations, firstly we did not analyze a morbidly obese subgroup (i.e., BMI > 40 kg/m^2^). As is the problem with many other studies, due to the low number of morbidly obese patients in our sample, an analysis with a morbidly obese subgroup would not have had sufficient power to detect any statistical significant differences. Secondly, our study sample had more females in the obese group than in the nonobese group. As there is some evidence that females report a lower function before surgery [28], future appropriately powered studies should repeat the analysis for males and females only or use gender as a covariate.

Compared to previous studies, strengths of our investigation include a comprehensive analysis of improvement in outcome scores and not just the latest follow-up score, correlation analysis and inclusion of patients undergoing surgery under a single surgeon and in a single institution with identical postoperative care plan to reduce heterogeneity of the sample.

## 5. Conclusion

With the increasing prevalence of obese patients in the TKA population, it is important to establish if these patients have results comparable with nonobese TKA or have to live with compromised results after surgery. The present study assesses the patients' perception of their outcomes which are important in clinical decision making as patients' concerns and priorities may be different from that assessed by health providers. The findings indicated that the patients perceived improvement in function and quality of life is not different between BMI groups based on a cut-off point of 30 kg/m^2^.

## Figures and Tables

**Table 1:** Mean (std.) of the demographics and number (%) of comorbidities for the two BMI groups and the total sample. *P* values of one-way ANOVA unless otherwise stated.

Variable	Total sample (*n* = 171)	Nonobese (*n* = 73)	Obese (*n* = 98)	*P*
Age (yrs)	66.7 (8.7)	68.0 (8.8)	65.7 (8.4)	0.089
BMI (kg/m^2^)	31.4 (5.6)	26.6 (2.1)	35.0 (4.7)	**<0.001**
Female *n* (%)^¥^	105 (60.3)	36 (48.0)	69 (69.7)	**0.002**
R.A *n* (%)	11 (6.3)	5 (6.6)	6 (6.1)	0.24
OKS (pre) (12–60)	43.3 (7.8)	41.1 (8.0)	44.7 (7.3)	**0.003**
SF12 PCS (pre)	29.0 (6.7)	28.8 (6.2)	30.0 (7.0)	0.833
SF12 MCS (pre)	49.4 (11.6)	51.1 (10.6)	48.3 (12.1)	0.118
Diabetes Mellitus *n* (%)^¥^	23 (13.2)	5 (6.6)	18 (18.2)	**0.015**
Hypertension *n* (%)^¥^	96 (55.2)	34 (45.3)	72 (72.7)	**<0.001**
Respiratory dis. (%)	33 (18.9)	15 (20)	18 (18.2)	0.16
Cardiac disease *n* (%)^¥^	22 (12.6)	8 (10.7)	14 (14.1)	0.15
Vascular disease *n* (%)^¥^	11 (6.3)	4 (5.3)	10 ( 10.1)	0.12
Previous TKR *n* (%)^¥^	28 (16.1)	14 (18.6)	14 (14.1)	0.12
Previous THR *n* (%)^¥^	22 (12.6)	8 (10.6)	14 (14.1)	0.15

^¥^Fisher's exact test, RA: rheumatoid arthritis, PCS: physical component summary of the SF12, and MCS: mental component summary of SF12.

**Table 2:** Mean (std.) of the values of the outcome measures for the two BMI groups at 6 months and one year. *P* values of the group and time effect (before surgery, 6 months and one year).

Variable	Nonobese (*n* = 25)	Obese (*n* = 39)	*P* value (group)	*P* value (time)
OKS 6 months	28.7 (10.4)	27.4 (9.0)	0.620	<0.001
OKS 1 year	27.1 (11.0)	25.7 (9.0)	0.328	
SF12 PCS 6 months	38.2 (10.4)	36.8 (10.5)	0.349	<0.001
SF12 PCS 1 year	41.1 (11.5)	36.6 (9.9)	0.310	
SF12 MCS 6 months	52.3 (9.6)	52.6 (9.5)	0.289	0.436
SF12 MCS 1 year	53.4 (8.2)	52.2 (8.9)	0.820	

PCS: physical component summary of the SF12, MCS: mental component summary of SF12.

**Table 3:** Mean (std.) of the change score data for the three BMI groups, means, and standard deviations.

Outcome	Nonobese (*n* = 25)	Obese (*n* = 39)	*P* value
OKS (pre-6mo)	−11.5 (10.9)	−15.3 (8.8)	0.129
OKS (pre-1yr)	−13.2 (9.9)	−17.0 (9.1)	0.120
SF12 PCS (pre-6mo)	8.4 (13.2)	6.9 (10.6)	0.603
SF12 PCS (pre-1yr)	11.3 (11.9)	6.7 (7.9)	0.061
SF12 MCS (pre-6mo)	0.5 (11.8)	3.1 (12.3)	0.233
SF12 MCS (pre-1yr)	0.7 (9.3)	2.7 (10.8)	0.428

PCS: physical component summary of the SF12, MCS: mental component summary of SF12.

**Table 4:** Pearson's correlation coefficients between BMI and the outcome measures before surgery, at 6 months and 1 year after surgery and between BMI and the change scores.

	OKS	SF12 (PCS)	SF12 (MCS)
Pre-op.	0.271*	−0.086	−0.101
6 months	0.115	0.143	−0.139
1 year	0.072	−0.206	−0.041
Pre-6m	−0.082	−0.117	−0.015
Pre-1yr	−0.194	−0.159	−0.068

*P < 0.01, PCS: physical component summary of the SF12, and MCS: mental component summary of SF12.

**Table 5:** Number (%) of postoperative complications and revisions in BMI groups.

	Nonobese (n = 73)	Obese (n = 98)	*P* value
Local complications	5 (6.7%)	11 (11%)	0.13
Systemic complications	6 (8%)	12 (12%)	0.14
Revisions	2 (3%)	3 (3%)	0.30
